# Keeping up with climate change: have Arctic arthropods reached their phenological limits?

**DOI:** 10.1098/rspb.2025.0350

**Published:** 2025-07-23

**Authors:** Hannah Sørine Gerlich, Martin Holmstrup, Niels Martin Schmidt, Albert B. Phillimore, Toke Thomas Høye

**Affiliations:** ^1^ Department of Ecoscience, Aarhus University, C.F. Møllers Allé 4-8, Aarhus C DK-8000, Denmark; ^2^ Arctic Research Centre, Aarhus University, Ole Worms Allé 1, Aarhus DK-8000, Denmark; ^3^ Department of Ecoscience, Aarhus University, Frederiksborgvej 399, Roskilde DK-4000, Denmark; ^4^ Institute of Ecology and Evolution, University of Edinburgh, Edinburgh EH9 3JT, UK

**Keywords:** arthropods, breakpoint phenological models, climate change, high Arctic, phenology

## Abstract

Many arthropods show earlier seasonal activity with warming, but these responses cannot continue indefinitely. Identifying such phenological thresholds is crucial for understanding limits to climate tracking and species persistence, but few studies test for breakpoints that may indicate physiological or ecological constraints. Using a 28-year time series, we examined breakpoint responses to snowmelt and temperature across 15 arthropod taxa in seven plots from high-Arctic Greenland, a region experiencing pronounced warming. Our meta-analysis found breakpoint responses in two of six phenological driver and event combinations: onset and peak activity advanced with earlier snowmelt until a threshold, beyond which the relationship levelled off. A breakpoint for peak activity in response to temperature disappeared when snowmelt was included in the model, underscoring the importance of considering several environmental cues to prevent incorrect inferences about plasticity limits. Most responses showed no evidence of a breakpoint in phenological sensitivity, instead exhibiting continued tracking of cues over the study period. Our findings suggest that while many Arctic arthropods remain responsive to climate change, some may be approaching limits, potentially altering ecological interactions and vulnerability to abiotic cues. Our findings highlight the need for broader assessments of phenological thresholds to refine predictions of species responses to environmental change.

## 1. Introduction

Shifts in the seasonal timing of life-history events are a well-documented ecological response to climate warming [1–3], especially in ectotherms like arthropods and plants [4–7]. When estimating relationships between phenology and environmental drivers many studies assume linearity between phenology and environmental drivers [8,9], with warmer springs often inducing earlier activity. While this assumption is often reasonable, continued climate change could cause linear responses to break down if plasticity in the timing of activities only keeps pace with environmental change within a range of abiotic conditions [10,11]. These limits have major implications for the ability of populations to track climate change [12] and maintain their phenological niche [13]. Ignoring potential nonlinear responses may obscure key insights into how organisms adjust to changing environments, limiting our understanding of plasticity mechanisms [14,15].

Among studies testing for nonlinear phenological responses, many still report linear advances in response to warming. Menzel *et al.* [12] and Jochner *et al.* [16] found consistent linear advances in European plant flowering over the 20th century. Similarly, Pearse *et al.* [17] observed linear shifts over a century in seven woody plant species in South Korea. However, nonlinear responses have been detected in certain taxa (e.g. [18–20]), such as North American tree swallows, which show constraints in advancing egg-laying dates [21]. Among arthropods, such responses are less studied, with most research focused on Lepidoptera. Long-term records of butterfly flight times in Britain report evidence that early-emerging species may be approaching the limits of phenological advancement, as their responses to warming have slowed in recent decades, with February temperatures now appearing more influential than those of March or April [22–24]. The scarcity of long-term datasets limits research on nonlinear responses in other arthropod taxa, creating a significant knowledge gap. This limits our ability to assess their climate sensitivity, roles in food webs [25,26], and to identify phenological thresholds [7,17].

The scarcity of long-term arthropod data extends to the Arctic, despite its unique value for studying climate impacts. With rapidly rising temperatures [27,28] and shifting snowmelt patterns [29,30], Arctic species may be among the first to reach the limits of phenological plasticity [31,32]. If physiological constraints impose thermal or photoperiodic thresholds, further phenological advancement may be restricted [33,34]. The ecosystem-wide monitoring programme initiated in 1996 at Zackenberg, northeast Greenland provides extensive time-series data for studying whether arthropods are approaching such limits. Observations suggest temperature and snowmelt are key cues for arthropod activity within their short seasonal window [35,36], but continued warming and earlier snowmelt may constrain their ability to track climate change.

**Figure 1.**
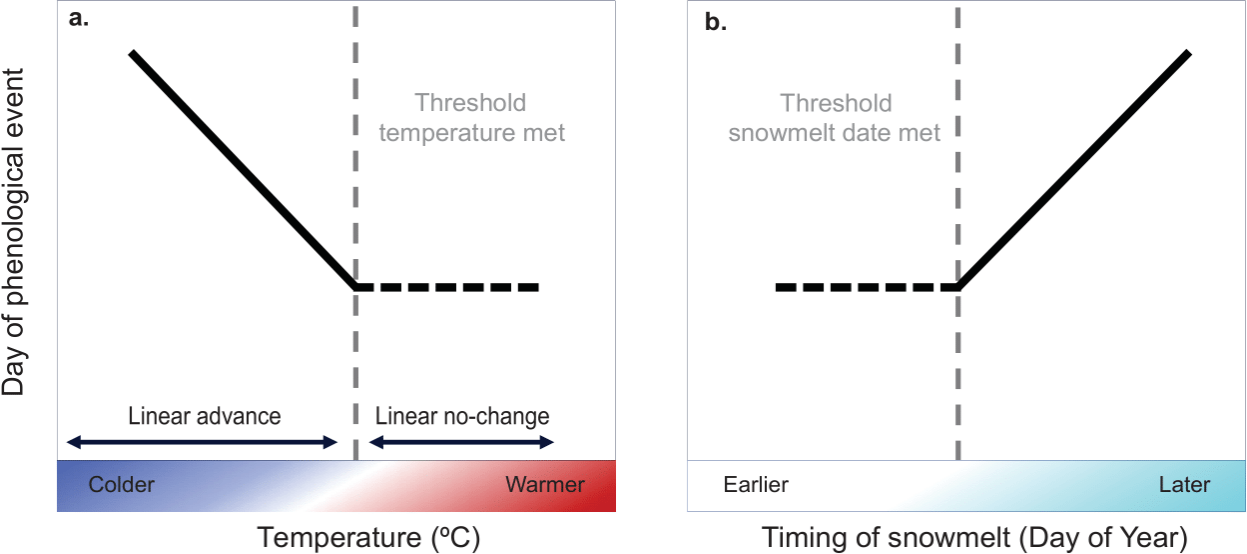
Examples of types of breakpoint-based thresholds in phenological responses of arthropod taxa to (a) temperature and (b) interannual variation in snowmelt. When a threshold is met, a change in slope is assumed (dashed vertical line) where the relationship between phenology and climate is continuous and piecewise linear. Taxa may show no response (slope not different from 0) on one side of the breakpoint (a,b) compared to a constant linear trend.

In the event that arthropods have reached a plasticity limit, we expect a threshold-like relationship between environmental variables and phenology, where the rate of change slows or plateaus beyond a certain point. This reaction norm can be identified using piecewise or breakpoint regression (figure 1a,b). Existence of a breakpoint indicates a shift in the slope of the linear regression, wherein on one side of this point, phenology ceases to respond or responds with a shallower gradient. Breakpoints can thus indicate reduced sensitivity at the warmer end of the temperature range or under earlier snowmelt dates [20,37]. However, apparent evidence of nonlinearity can also arise as an artefact through incorrect specification of the statistical model. For instance, where there is a nonlinear relationship between the focal climate predictor and the actual cue, or where the focal climate predictor is correlated with an actual cue that has a nonlinear effect (e.g. [38,39]). While studies exploring nonlinearity or other types of phenology–climate relationships often neglect the inclusion of multiple climate predictors, incorporating several key climate variables provides a more robust approach to detecting causal effects and minimizes the risk of misinterpreting nonlinear responses as artefacts of model specification—an approach emphasized in this study.

Previous work at Zackenberg has examined phenological limits in plants [20], and analyses using ≥10 years of data suggest declining phenological sensitivities to temperature and snowmelt for arthropods, plants, and birds [20,40]. To test for similar patterns in activity timing of arthropods, generalized additive modelling (GAM) was applied to phenology-climate relationships [40]. Nonlinear trends consistent with plasticity limits were detected in response to temperature for four of 12 taxa (Muscidae, Sciaridae, Nymphalidae and Lycosidae) and to snowmelt for five taxa (Chironomidae, Sciaridae, Collembola, Acari and Coccoidea; figure 1). While GAMs are effective for visualizing nonlinear relationships [41], they do not estimate the point or extent of sensitivity change over time.

In comparison with GAMs, breakpoint regression estimates slope changes in phenological trends [42], enabling detection of threshold-like responses that may indicate plasticity limits. Breakpoint regression also yields comparable and easily interpretable effect sizes, suitable for meta-analysis [43,44]. This is particularly valuable when working with small datasets that individually offer limited statistical power, as is the case for the Zackenberg phenological time series. Unlike the typical use of meta-analyses that synthesize results across different studies to estimate an overall effect size (e.g. how a treatment influences a species across studies), we apply a meta-analytic framework within a single study to examine the average evidence for breakpoint responses to environmental drivers across taxa and habitats. This allows us to identify common breakpoint response shapes and quantify variation across taxa and across different plots and habitats.

Specifically, we assess whether arthropod phenology shows breakpoint responses to temperature and snowmelt timing using 28 years (1996–2023) of annual records of onset, peak and end of activity at Zackenberg. This dual perspective—examining both overarching patterns and their underlying variability—provides a robust framework for understanding potential limits in phenological plasticity in a rapidly changing Arctic.

We address the following questions. (i) In general, do arthropod taxa show breakpoint phenological responses to temperature or timing of snowmelt? (ii) Do the breakpoint responses to a focal climate variable remain even when controlling for the effect of the other main climate driver (i.e. temperature or snowmelt)? (iii) If there is evidence of breakpoints, do they cluster around similar climate values across taxa or span a broad range of conditions? (iv) Does the type of breakpoint model vary among taxa and/or habitats? For instance, earlier timings of early-active taxa may be more prone to reaching breakpoints as illustrated in figure 1, compared with late-active taxa.

## 2. Material and methods

### (a) Study site and arthropod sampling

Arthropods and climate data were collected at the Zackenberg Research Station, in high-Arctic Northeast Greenland (74°28′ N; 20°34′ W) as part of the Greenland Ecosystem Monitoring Program since 1996. Arthropods were sampled each growing season in seven plots (plots 1–7) across five habitats. Plots 2–7 included eight yellow pitfall traps (1997–2006) later reduced to four pitfall traps (2007–2023), and plot 1 included two window traps (flight-interception traps). Plot 6 was closed in 1999 but reopened in 2016. Traps were emptied weekly on fixed dates from snowmelt (usually from late May to early June) until freezing. Specimens were sorted to family for spiders and most insects, superfamily for Aphidoidea, Chalcidoidea and Coccoidea and subclass for other arthropods, and subsequently counted. The data are publicly available at http://data.g-e-m.dk (https://doi.org/10.17897/KBN7-WP73).

Plots represented pond (plot 1), wet fen (plot 2), mesic heath (plots 3 and 4), snow bed (plot 6) and arid heath (plots 5 and 7) differing in plant community composition, soil moisture, and snowmelt timing. Further plot details can be found in Schmidt *et al.* [45] and Høye & Forchhammer [36]; abundance estimates in Høye *et al.* [46].

### (b) Climate variables

We used air temperature and snowmelt timing to determine the effect of climate on activity. A climate tower located centrally and within 600 m from all plots measured air temperature at 2 m height on an hourly basis throughout the entire study period (downloaded: 13 January 2025). From this data, we computed temperature predictors for each phenological event separately. Temperature in the period before emergence is closely related to arthropod development [47] and is therefore expected to be a good predictor of adult arthropod activity. To capture lower-end variability and reflect critical fluctuations affecting development, we calculated the mean phenological date and then subtracted one s.d. before averaging temperatures over the preceding 30 days.

Snowmelt timing was estimated as the date when snow depth dropped below 10 cm, measured by an automatic snow depth sensor situated by the meteorological station [48]. In 2009, 2013 and 2019, snow accumulation was very limited, and the snow depth analyser was not sufficient for accurate snowmelt estimation. In these cases, we used soil temperature data (averaged from 0, 5 and 10 cm depth) to estimate more reliable snow melting dates. We identified the time period towards the end of the winter when ground temperatures were stable near 0°C and subsequently started fluctuating as the snow cover disappeared, known as the zero-curtain window. This method, as outlined in Rixen *et al.* [30], is considered more reliable in years with limited snow accumulation because soil temperatures provide a good indication of when the snow has fully melted. We refer to Gerlich *et al.* [49] for a detailed explanation of the calculations.

For each taxon-by-plot analysis, we assessed the degree of multicollinearity of climate predictors by calculating the variance inflation factors (VIFs) prior to executing model runs. The average VIF for air temperature and snowmelt timing was 1.48 with max 4.60. While this level of collinearity is generally not considered problematic (VIF < 5), we tested model sensitivity by running them with and without both predictors. The results showed consistent trends across model runs, indicating that the models are robust to multicollinearity. Therefore, we consider including both predictors reasonable.

### (c) Calculating phenological events

Annual onset, peak and end of activity of arthropod taxa across each habitat were calculated using GAMs on the abundance per trap per day for each plot in each season, following [49]. We fitted smoothers for day of year using a Poisson distribution with *k* = 4 (basis dimensions) to ensure appropriate smoothing and a log link function, with the package ‘*mgcv’* v. 1.8-40 [50]. The basis type used in the smoothers was cubic regression splines, which allow for flexible, nonlinear relationships between time and abundance. We did not explicitly check the residuals for autocorrelation, as the flexibility of the GAM framework, including the smooth terms, accounts for complex nonlinear patterns in the data. We restricted our analyses to years where at least 50 individuals of a given taxon were caught in total across all habitat plots. Also, we required that all three phenological events could be calculated (the taxa must be present in at least two weeks) and a plausible seasonal curve could be generated (for example, if sampling began or ended during peak abundance of some early- or late-emerging taxa, the phenological events could not be calculated accurately, and therefore, these years were omitted). As most high-Arctic arthropods are univoltine, an approximately symmetric distribution in seasonal activity among taxa was found. If asymmetric phenological curves or multimodal distributions were found, we excluded the taxon in the specific year. When filtering for these criteria, 1319 years-by-taxon-by-plot data points were included in the study. Annual onset, peak and end of the activity season were then calculated as the day at which 10%, 50% and 90% of cumulative abundance (area under the curve) were reached, respectively.

### (d) Statistical analysis

#### (i) Breakpoint regression analyses

We used a piecewise linear regression approach to assess relationships between phenological events and climate variables for each taxon-by-plot combination, alongside linear models for comparison. We fitted separate models for each arthropod taxon, plot and phenological response, using either snowmelt timing or temperature as continuous predictors. In total, we analysed 63 time series per phenological event (corresponding to the number of taxon-by-plot combinations meeting our inclusion criteria). We also tested models with both predictors, but in these cases, allowing only one predictor at a time to have a breakpoint. Our motivation for including two climate predictors and only one breakpoint was to test the hypothesis that evidence for a breakpoint was dependent on the inclusion/exclusion of a correlated but causal climate variable. E.g., if snowmelt is a primary driver, including it as a covariate could reduce or eliminate evidence for a temperature breakpoint.

The breakpoint model was structured as follows:


$$y\;=\;\beta_{0}\;+\;\beta_{1}x_{1}\;+\;\beta_{2}\left(x_{1}\;-\;k\right)\left(x_{1}\geq k\right)\;+\;e$$

where


$$\left(x_{1}-\;k\right)\;=\;\left\{\begin{array}{ll} 0, & if\;x<k\\ x-k, & if\;x\;\geq k \end{array}\right\}$$

Here, *β_0_* is the intercept, *β_1_* is the slope of the first linear segment (we refer to this as slope 1), *β_2_* is the difference between the slope of the first and second linear segment (referred to as Δ slope), *k* is the breakpoint (position of change in slope), which is assumed to be at the same position on x as one of the data points, and *e* is the random independent error. The breakpoint was found using a maximum likelihood approach where the best breakpoint was the one that maximized the likelihood of the data. When the breakpoint was determined, we computed the regression coefficients of the first linear segment and the Δ slope for each model. We used simulations to test the type I error rate and power of this approach (electronic supplementary material, figure S1.1 and S1.3). Simulations revealed that the standard error of the Δ slope estimate was substantially underestimated (electronic supplementary material, figure S1.2), and the type I error rate of the Δ slope test thus greatly elevated. This is a result of the multiple-testing aspect of locating a breakpoint not being appropriately accounted for. To remedy this, we fitted a model with no breakpoint (i.e. the null) and then simulated response data under this model 1000 times. For each simulation, we fitted the linear model with a single slope and a second model where we had identified the best breakpoint and the difference between the two slopes. Then, to obtain a *p* value for the Δ slope, we compared the observed log-likelihood difference between the breakpoint model and single linear slope model to the distribution of log-likelihood difference values generated from simulations under the null. The *p* value was calculated as the proportion of the null simulated values that exceeded the observed value. Simulations showed that this approach to obtaining *p* values yielded appropriate type I errors (electronic supplementary material, figure S1.1). For the meta-analysis, we also required estimates of parameter uncertainty for slope 1 and Δ slope. We used the s.d. of slope 1 and Δ slope estimates across the null simulations to obtain standard errors for slope 1 and Δ slope.

### (e) Meta-analysis

The meta-analysis was performed using the package ‘*metafor*’ v. 4.8-0 in R [51].

Meta-regression analysis provides an efficient and powerful statistical tool to identify average effects across time series [42,52].

To estimate the average *β*₁ and *β*₂ across the arthropod community and assess whether taxa exhibit breakpoint phenological responses to temperature or timing of snowmelt, we included the slope estimates for each time series and their respective squared standard errors in a random-effects meta-regression model [51]. Random intercepts were included for arthropod taxon, plot and taxon-by-plot combinations to account for non-independence. To assess whether breakpoint responses persisted when accounting for both climate drivers, we fit four models per phenological event (onset, peak, and end): (1) snowmelt timing as the sole predictor, (2) temperature as the sole predictor, (3) snowmelt as the main predictor while controlling for temperature, and (4) temperature as the main predictor while controlling for snowmelt. To test whether slope estimates changed when accounting for both predictors, we performed a meta-regression with model type (single versus combined predictor) as a moderator. Here, the response variable was either the Δ slope or slope 1 estimates, with squared standard errors as the variance term. Statistical inference focused primarily on the effect size (Δ slope or slope 1) and its 95% confidence intervals, where intervals not spanning zero indicated a significant response. Model fit was evaluated by examining random effect variance components, assessing heterogeneity using *Q*-tests and inspecting residual distributions to identify potential patterns or deviations. To assess whether breakpoint models outperformed linear models in relative fit and explanatory power, we compared *R*^²^ values between approaches.

Finally, to evaluate variation in breakpoint responses across taxa and habitats, we conducted likelihood ratio tests (LRTs) comparing models with and without these random terms. We also ran simplified models without random effects to assess the relative contribution of taxon- and habitat-level variation.

## 3. Results

### (a) Phenological responses to timing of snowmelt

#### (i) Do arthropod taxa show breakpoint phenological responses to timing of snowmelt?

**Figure 2.**
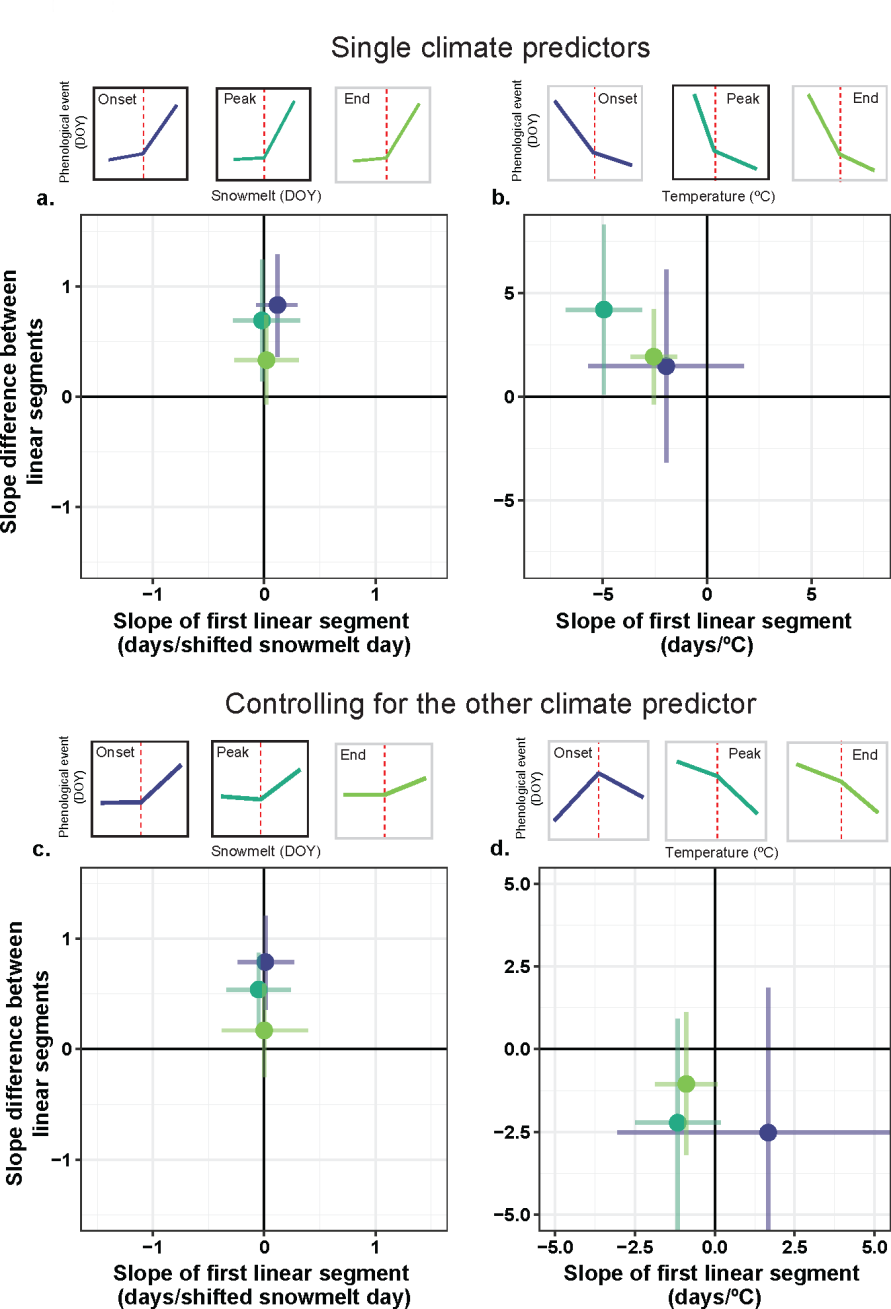
Types of breakpoint responses to snowmelt and temperature on average in the arthropod community, across onset, peak and end of seasonal activity, based on meta-analysis results summarized in table 1. Panels (a–d) plot the estimated average Δ slope between the first and second linear segments against the estimated average slope of the first segment. To illustrate these responses, the smaller panels above each main panel depict the type of breakpoint response for each phenological event, differentiated by colour. Black panels indicate significant breakpoint models (where Δ slope was significantly different from zero), and grey panels indicate non-significant models. Panels (a) and (b) display responses to snowmelt and temperature as single predictors, while panels (c) and (d) show responses when controlling for the other predictor. Error bars represent 95% confidence intervals. Point estimates for all taxa and plots can be seen in electronic supplementary material, figures S2.4 and S2.6.

Our meta-analysis confirmed breakpoint phenological responses to snowmelt timing. The most common trend was no response to very early snowmelt, as indicated by a non-significant first-segment slope (figure 2a and table 1), followed by delayed activity with later snowmelt, reflected in a positive Δ slope (figure 2a and table 1). This threshold effect was strongest for onset and peak of activity, where Δ slopes were significantly >0 (table 1). In years where snowmelt was after the threshold date, onset advanced by an average rate of approximately 0.95 days and peak by approximately 0.71 days for every day that snow melted earlier. These estimates are the slope of the second linear segment, derived from the model’s first-segment slope and slope difference (table 1), and do not have associated standard errors. In contrast, when snowmelt occurred before the threshold, the average advance was much weaker, at 0.12 ± 0.09 days for onset and 0.02 ± 0.15 for peak of activity. While both breakpoint and linear models fit the data well based on *R*^2^ values (electronic supplementary material, figure S2.1), the breakpoint model provided a better fit (electronic supplementary material, figure S2.2), highlighting that arthropod phenological responses to snowmelt are often nonlinear and best described by threshold-dependent shifts. For end of activity, the Δ slope was also positive but not significant, suggesting a weaker or more variable response (table 1).

**Table 1.** Summary of meta-analysis model outputs for the first slope and Δ slope from breakpoint regressions of arthropod onset, peak and end of activity in response to climate variables: timing of snowmelt (snowmelt) and air temperature (temperature) separately and in combination. The continuous climate variable that was subject to the breakpoint analysis is indicated in the ‘predictor’ column. Significant estimates (*p* < 0.05) are shown in bold. Estimates from corresponding simple linear regression models are provided in electronic supplementary material, table S2.2.

model	predictor	phenological event	slope 1			slope difference		
			**Est.**	se	CI	**Est.**	se	CI
snowmelt	snowmelt	onset	0.12	0.09	−0.07, 0.30	**0.83**	0.24	0.36, 1.29
		peak	0.02	0.15	−0.28, 0.32	**0.69**	0.28	0.14, 1.24
		end	0.02	0.15	−0.27, 0.31	0.33	0.21	−0.07, 0.74
snowmelt + temperature	snowmelt	onset	0.01	0.12	−0.24, 0.27	**0.79**	0.22	0.36, 1.21
		peak	−0.05	0.15	−0.34, 0.24	**0.54**	0.17	0.21, 0.87
		end	0.00	0.20	−0.38, 0.39	0.17	0.21	−0.25, 0.59
temperature	temperature	onset	−1.96	1.90	−5.68, 1.77	1.47	2.38	−3.19, 6.12
		peak	**−4.95**	0.92	−6.76, −3.14	**4.20**	2.09	0.11, 8.29
		end	**−2.56**	0.57	−3.67, −1.46	1.93	1.17	−0.36, 4.21
temperature + snowmelt	temperature	onset	1.67	2.41	−3.05, 6.39	−2.52	2.24	−6.90, 1.86
		peak	−1.16	0.68	−2.49, 0.18	−2.22	1.60	−5.35, 0.92
		end	−0.89	0.50	−1.86, 0.08	−1.05	1.10	−3.20, 1.11

#### (ii) Do the breakpoint responses to snowmelt disappear when controlling for the effect of temperature?

Snowmelt timing remained a strong predictor of all three phenological events, even after controlling for temperature effects, with trends similar to snowmelt-only models (table 1 and figure 2c). This supports snowmelt as a primary phenological cue, largely independent of temperature. In years with later snowmelt, arthropods advanced their onset of activity by approximately 0.80 days and peak activity by approximately 0.49 days for each day of earlier snowmelt. These estimates, derived from the second segment slope, lack standard errors. Slope estimates were largely consistent between the snowmelt-only model and the model that included temperature (electronic supplementary material, table S2.1), indicating the minimal influence of temperature on the snowmelt–phenology relationship. In particular, Δ slope did not differ significantly between models, and the first-segment slope remained largely stable, except for onset, where the slope shifted from 0.12 ± 0.09 in the snowmelt-only model but approached zero (0.01 ± 0.12) when temperature was included. Breakpoint models continued to outperform linear models (electronic supplementary material, figures S2.1 and S2.2), reinforcing the presence of nonlinear, threshold-like responses to snowmelt.

#### (iii) Do all taxa reach a breakpoint at similar snowmelt dates?

**Figure 3.**
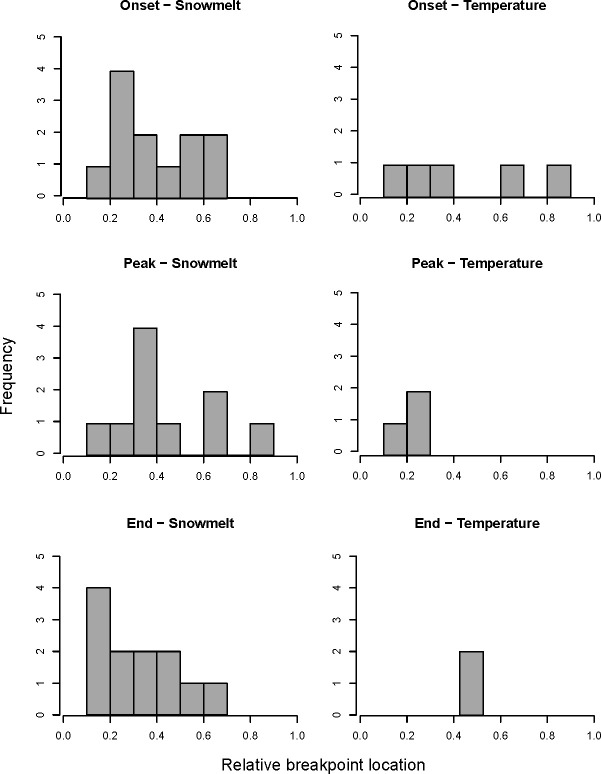
Frequency in location of estimated breakpoints from breakpoint regression models of onset, peak and end of activity for all arthropod taxa and plot combinations with temperature and timing of snowmelt as climate predictors. Only breakpoints from significant breakpoint models controlling for the other predictor are shown. The range of *x*-values (breakpoint location) has been normalized for all taxa and habitat combinations to range between 0 (minimum temperature or earliest timing of snowmelt experienced by a taxon) and 1 (maximum temperature or latest timing of snowmelt).

Breakpoints for onset and peak occurred around day 171 ± 9.08 (20 June) and 167 ± 7.25 (15 June), respectively, closely aligning with estimates from snowmelt-only models, with distinct shifts occurring at day 170 ± 8.78 (19 June) for the onset and day 166 ± 7.26 (14 June) for the peak of activity. However, breakpoint dates varied across taxa and were distributed across a range of snowmelt dates, both in models with and without temperature, highlighting that not all taxa reached breakpoints at the same point along the snowmelt gradient (figure 3 and electronic supplementary material, figure S2.5).

#### (iv) Does the type of breakpoint model vary among taxa and/or habitats?

**Figure 4.**
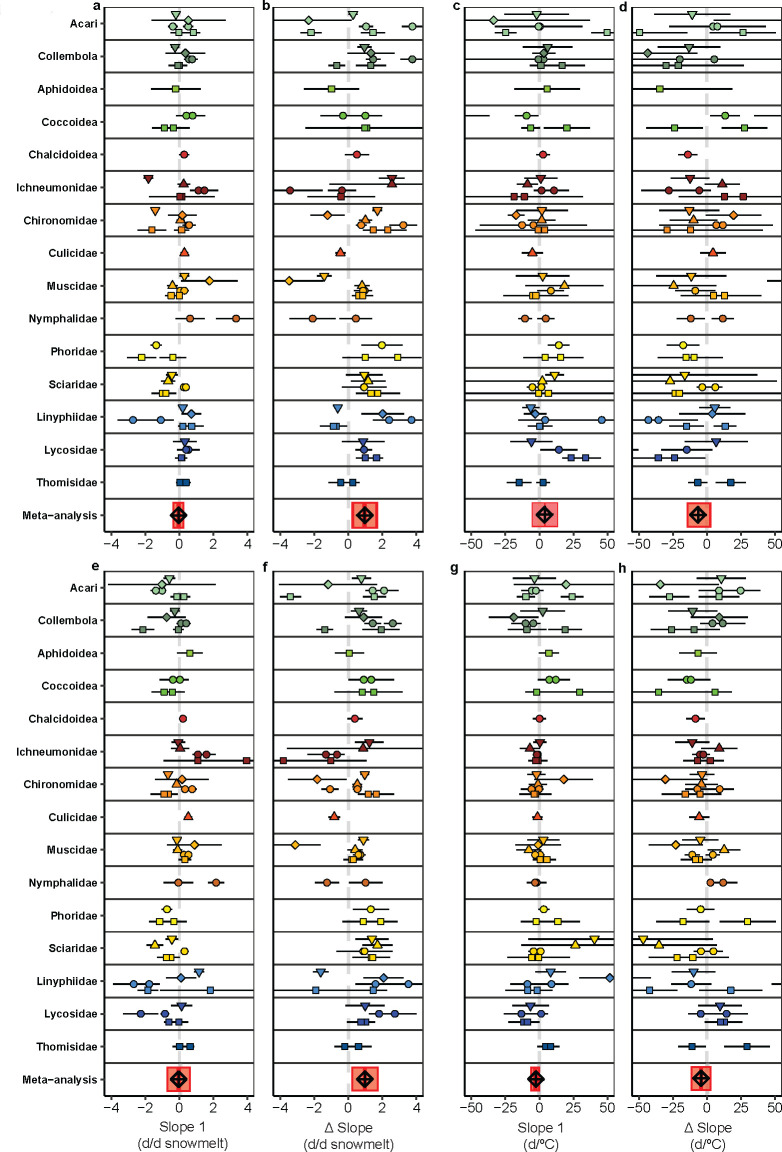
Forest plot showing slope estimates for the first linear segment (a,c,e,g) and Δ slope (b,d,f,h) for the onset and peak of arthropod activity in response to snowmelt (onset: (a,b) and peak: (e,f)) and temperature (onset: (c,d) and peak: (g,h)), while controlling for the other predictor. Results are presented for all taxon-by-plot combinations included in the study. Error bars indicate the standard error of the mean. The final row in each panel includes the meta-analysis average estimates with confidence intervals. Similar figures for the end of activity can be found in the electronic supplementary material, figure S2.7.

Taxon and habitat-level differences significantly contributed to variation in breakpoint responses to snowmelt timing (figure 4). Meta-regression models with random effects for taxon and plot, showed that both factors contributed to differences in Δ slope across phenological events (taxon: onset LRT = 64.58, *p* < 0.001; peak LRT = 42.90, *p* < 0.001; end LRT = 80.02, *p* < 0.001; plot: onset LRT = 47.99, *p* < 0.001; peak LRT = 8.49, *p* < 0.01; end LRT = 9.86, *p* < 0.01; electronic supplementary material, tables S3.1 and S3.2). Breakpoint trends closely mirrored those observed in models with snowmelt as a single predictor (compare figure 2a,c), with taxon-level variation explaining most of the observed heterogeneity, with smaller contributions from plot-level variability. E.g., early and late-active taxa showed distinct responses: some shifted rapidly with earlier snowmelt (e.g. Ichneumonidae in wet fen and Phoridae in mesic heath), while others responded more slowly or not at all (e.g. Linyphiidae and Chironomidae in mesic heath; electronic supplementary material, tables S3.1 and S3.2). These results indicate that while snowmelt timing is a dominant driver, responses are modulated by species traits and habitat context.

### (b) Phenological responses to temperature

#### (i) Do arthropod taxa show breakpoint phenological responses to temperature?

Breakpoint responses to temperature were detected, with significant Δ slopes for peak activity, while similar but weaker trends were observed for onset and end of activity (table 1). The most common pattern was a negative relationship between temperature and phenology at colder ranges, with peak activity advancing by −4.95 ± 0.92 days per °C up to a threshold temperature, above which further shifts were minimal or absent (table 1 and figure 2b). For onset and end of activity, no significant breakpoint was found. Instead, phenology followed a consistent negative linear trend across the full temperature range, with activity advancing by −2.54 ± 0.62 days per °C for onset and −1.59 ± 0.50 days per °C for end (electronic supplementary material, table S2.2). *R*² comparisons between breakpoint and linear models indicated that the breakpoint model did not provide a distinctly better fit (electronic supplementary material, figure S2.2).

#### (ii) Do the breakpoint responses to temperature disappear when controlling for the effect of snowmelt?

We found little support for breakpoint models after controlling for timing of snowmelt with the Δ slope estimate being much closer to zero (table 1), and temperature–phenology relationships became shallower (table 1 and figure 2d). For onset, the previously negative slope shifted slightly positive, though confidence intervals overlapped zero (table 1). Meta-regression comparing models with and without snowmelt showed significant differences in the slope of the first linear segment and Δ slope for peak and end of activity (electronic supplementary material, table S2.1). Including snowmelt reduced the magnitude of temperature effects, suggesting that the observed breakpoints were largely driven by snowmelt rather than temperature itself. This highlights the dominant role of snowmelt timing in shaping phenological responses. Linear models better captured temperature effects across the full gradient (electronic supplementary material, figure S2.2): −1.60 ± 0.80 days per °C for onset, −2.08 ± 0.47 days per °C for peak and −0.82 ± 0.30 days per °C for end (electronic supplementary material, table S2.2). Thus, we find no strong evidence of threshold-like responses to temperature once snowmelt was considered, reinforcing the primary role of snowmelt in driving phenological shifts in the Zackenberg arthropod community.

#### (iii) Do all taxa reach a breakpoint at similar temperatures?

A distinct shift in peak activity occured at an average threshold temperature of 3.58 ± 1.91°C when temperature was the sole predictor. However, significant breakpoints in taxon-by-plot combinations were generally observed at lower average temperatures, ranging from 0.15 to 2.73°C (electronic supplementary material, figure S2.5).

#### (iv) Does the type of breakpoint model vary among taxa and/or habitats?

Taxon and habitat had little impact on breakpoint responses to temperature. LRTs indicated that including random effects for taxon or plot rarely improved model fit (electronic supplementary material, tables S3.1 and S3.2). Still, some variation was observed among individual taxon-by-plot combinations (figure 4), with taxa like Chironomidae, Chalcidoidea, Acari and Lycosidae exhibiting significant breakpoint responses in the onset of activity, with a positive relationship to temperature in the first linear segment, followed by no response or a slightly negative response beyond the breakpoint—mirroring the general community trend (table 1 and figure 2d).

## 4. Discussion

For four of six phenology-climate combinations considered for this high-Arctic arthropod community, we found overall support for linear advances in activity in response to temperature and snowmelt timing, suggesting that Arctic arthropods, in general, are currently capable of at least partially tracking changes in environmental conditions. However, for onset and peak of activity in response to snowmelt date, we observed breakpoint responses, suggesting that some aspects of phenology for the average arthropod are approaching their limits to advance in response to earlier snowmelt. Evidence of a breakpoint response in peak phenology to temperature was also found, but this effect was not robust to controlling for the timing of snowmelt in the model, indicating that failure to consider multiple environmental cues could lead to incorrect inferences about limits to phenological responses. While sampling variance contributes substantially to among time-series variation in slope estimates (e.g. see the slope variation in figure 4), after controlling for this, our meta-analysis found variations in the type of breakpoint phenological response among arthropod taxa and between the same arthropod taxon from different habitats.

### (a) Breakpoint responses to timing of snowmelt

Indications of nonlinear phenological responses of arthropod taxa to snowmelt in the high-Arctic have previously been reported [40]. We extend these findings by showing that, on average, for every day snow melted earlier, the onset of arthropod activity advanced by approximately 0.80 days and the peak by approximately 0.49 days until a threshold snowmelt date was reached at day 170 ± 8.78 (19 June) for the onset and day 166 ± 7.26 (14 June) for the peak of activity. In years when the snow melted before this threshold date, there was no evidence of a significant phenological response to earlier snowmelt at the community level. From this, we infer that high-Arctic arthropods may be approaching a limit in their ability to track increasingly earlier snowmelt. A similar study on six plant species at Zackenberg, based on 16 years of data [20], found that at least one species (*Papaver radicatum*) exhibited a comparable breakpoint response, advancing its flowering by an average of 1.11 ± 0.02 days for every day snow melted earlier, until a threshold around 15 June. This suggests that nonlinear phenological shifts in this location are not confined to arthropods. Despite differences in life histories and ecological roles, various taxa, including arthropods, plants and birds [40], may share common limits in their ability to advance phenology beyond a certain threshold.

### (b) Causes for constraints on phenological plasticity

Our findings suggest that arthropod phenology may be reaching limits in tracking earlier snowmelt, potentially constrained by other abiotic cues such as photoperiod [39]. As a key regulator of diapause termination, photoperiod may prevent emergence if critical thresholds are not met [53], especially in highly seasonal environments where it helps organisms avoid development during unusually warm spells too early in the year [33]. For soil microarthropods, photoperiod may prove to be a less relevant cue, as they do not enter dormant diapause stages and become surface-active as soon as temperature and moisture permit. Earlier snowmelt could expose arthropods to frost damage and desiccation, especially if delayed emergence reduces the insulating effect of snow cover [54]. Climate-induced variability in snow cover further complicates predictions [30,49]. Some species may delay development or extend diapause [55], as a plastic response to avoid unfavourable conditions. However, reduced snow cover might further complicate matters by reducing moisture availability, increasing desiccation risk for early season stages, especially in soil microarthropods and Diptera larvae. This could drive selection for more resilient populations while reducing survival among less plastic species. Future research should explore whether mismatches between snowmelt and arthropod development are already occurring using long-term data and fine-scale microclimate measurements. Experimental snowmelt manipulations could clarify if delayed emergence is driven by photoperiod constraints or ecological trade-offs linked to early season conditions [56,57].

### (c) No evidence for breakpoint responses to temperature

Temperature-driven shifts in phenology varied depending on whether snowmelt was included in the model. When temperature was considered alone, the average community peak phenology advanced by 4.95 days per °C until reaching a threshold, after which the rate slowed to 0.75 days per °C. However, once snowmelt was accounted for, the effect dropped to 1.16 days per °C before the threshold, suggesting that some of the effects that had been attributed to temperature when considered in isolation may reflect temperature-snowmelt correlations [38]. As such, temperature alone may not capture the abiotic conditions affecting the phenology of Arctic arthropods, and temperature-only models may provide inaccurate predictions. Instead, we observed consistent but shallow linear responses, indicating no clear limits to temperature sensitivity within the range currently experienced. These findings underscore the importance of including multiple environmental drivers, depending on the bioclimatic region, in phenology studies [39,58,59].

### (d) Methodological considerations

The stronger role of snowmelt than temperature in shaping arthropod phenology may partly reflect limitations in our temperature metric and taxonomic resolution. Many arthropods respond more strongly to cumulative temperature exposure, rather than 30 day means before a phenological event. Alternative semi-mechanistic approaches to modelling temperature–phenology relationships, such as growing degree days or considering the temperature between snowmelt until emergence event [60,61], might better capture species-specific thermal sensitivities. Also, using a single temperature value across entire families may mask interspecific variation, making snowmelt appear as the dominant driver. However, refining temperature predictors was constrained by the coarse resolution of our dataset. Our snowmelt metric may also influence results: we defined onset as the calendar day of first activity, which captures both direct and indirect snowmelt effects. Expressing phenology as days after snowmelt could isolate snowmelt effects, but would flatten slopes in linear models and obscure nonlinear patterns. Future studies could explore alternative formulations to better disentangle the roles of snowmelt and temperature.

Our breakpoint regression approach assumes a sudden shift in phenological sensitivity to environmental conditions. However, responses may change more gradually as species approach physiological or ecological limits due to developmental constraints, the influence of additional environmental cues or ecological trade-offs associated with too early activity. Other nonlinear models, such as logistic or sigmoidal functions [8,16,62], may better capture these dynamics. Another way to assess shifts in sensitivity is to examine variations in arthropod activity before and after the breakpoint. If the breakpoint represents a meaningful ecological threshold, we expect phenological timing to track snowmelt closely when it occurs later than the threshold, resulting in greater year-to-year variability. In contrast, when snowmelt occurs earlier than the threshold, responses may become more consistent due to constraints like photoperiod [20,26]. Future studies could validate this by quantifying variance in phenology across different snowmelt regimes or applying models that allow more gradual changes. Nonetheless, breakpoint regression remains a powerful tool for detecting critical thresholds in phenological shifts and identifying potential limits to plasticity.

### (e) Interspecific variation and implications for trophic interactions

Species differ in their sensitivity to climate drivers based on life history traits [63], resource availability [4], and adaptative strategies [64]. Our analyses revealed marked variation in Δ slope estimates across taxa and plots, suggesting diverse breakpoint responses within the community. Although not formally tested, early-season taxa, such as Lycosidae and Collembola, appeared less sensitive to early snowmelt in this study, suggesting that their ability to track climate-driven changes may be altered. Some species within these taxa may begin activity before snowmelt, even though snowmelt is generally a strong cue for emergence [65,66]. However, because traps can only be deployed once sites are snow-free, early activity may go undetected. Despite this potential limitation, divergent responses between early or late-active taxa could disrupt trophic interactions that rely on temporal overlap [67].

Pooling species into larger taxonomic units, such as family level, can obscure species-specific responses [68], especially for large and diverse groups such as arthropods. At the family level, we cannot disentangle the effects of shifts in species composition and changes in individual species phenology. This could affect our results, as advanced emergence in early snow-melting years might reflect either a general advancement in species' phenology or early-emerging species becoming more common. Species level studies are needed to discern which species are closest to their phenological limits. Nonetheless, our detection of breakpoint responses in the onset of activity to snowmelt timing at the family level already implies a community-wide shift in climate sensitivity. Given a relatively widespread, strong phylogenetic signal in phenological plasticity [64,69], family level patterns may reflect species level trends. Our community-level approach also provides insights into how phenological shifts may affect energy flow and food webs dynamics in Arctic ecosystems [70].

## 5. Conclusions

With continued climate change in the Arctic, the assumption of linear phenology-climate relationships is likely to break down. This study highlights the importance of investigating nonlinear phenological responses to multiple climate predictors and the value of long-term datasets to detect these patterns. The discovery of breakpoint responses to snowmelt timing in high-Arctic arthropods suggests that these species may not be able to shift their activity earlier under future earlier snowmelt. Variation in responses across taxa underscores taxon-specific climate sensitivity. Importantly, multiple cues affect phenology–climate relationships, contributing to the complexity of modelling responses to climate change. Therefore, we encourage wider testing of nonlinear relationships and interactions between climate predictors in phenological studies.

## Data Availability

We commit to public data access for scientific reproducibility and transparency. All data used in this study are available from Dryad [71]. This includes phenology and climate data. The R script to replicate all results and figures is available from Zenodo [72]. Supplementary material is available online [73].

## Authors' Notes

Throughout the study period, all animals were collected in accordance with annual permits and regulations from the government of Greenland under the non-exclusive licence no. for utilization of Greenland genetic resources, granted to Arctic Research Center, Aarhus University (e.g. journal nos. G19-006, G21-005).
